# EASIX and Arterial stiffness in relation to nocturnal blood pressure patterns in newly diagnosed hypertension

**DOI:** 10.1038/s41371-026-01160-7

**Published:** 2026-05-19

**Authors:** Muhammed Ulvi Yalcin, Kadri Murat Gurses, Halil Ozalp, Muhammet Salih Ates, Abdullah Tuncez, Huseyin Tezcan, Yasin Ozen, Tugay Dedebali, Mustafa Abdullah Kebude, Bulent Behlul Altunkeser

**Affiliations:** 1 Department of Cardiology, Selcuk University Faculty of Medicine, Konya, Turkey; 2 Department of Cardiology, Ahi Evran University Faculty of Medicine, Kırsehir, Turkey

**Keywords:** Prognosis, Risk factors

## Abstract

Non-dipper hypertension is associated with increased cardiovascular risk and target organ damage. Although endothelial dysfunction and arterial stiffness contribute to impaired nocturnal blood pressure decline, their relationship with the Endothelial Activation and Stress Index (EASIX) remains unclear. This study aimed to evaluate the association between nocturnal dipping status, EASIX score, and arterial stiffness. This retrospective study included 163 newly diagnosed hypertensive patients who underwent 24-hour ambulatory blood pressure monitoring and were classified as dipper or non-dipper based on nocturnal systolic blood pressure decline. Arterial stiffness was assessed using pulse wave velocity (PWV), and EASIX was calculated from lactate dehydrogenase, creatinine, and platelet count. Logistic regression analysis was performed to identify independent predictors of non-dipper status, while receiver operating characteristic analysis evaluated the diagnostic performance of EASIX, PWV, and their combination. Seventy-two patients were classified as non-dippers and 91 as dippers. Non-dipper patients had significantly higher EASIX scores (0.73 vs. 0.52, *p* < 0.001) and PWV values (7.92 vs. 7.05 m/s, *p* = 0.003). In multivariable analysis, EASIX (OR: 2.553, 95% CI: 1.568–4.156, *p* < 0.001) and PWV (OR: 1.358, 95% CI: 1.090–1.691, *p* = 0.006) remained independently associated with non-dipper status. The combined model demonstrated better discrimination (AUC: 0.755) than EASIX (0.723) or PWV (0.625) alone. Higher EASIX scores and increased arterial stiffness were independently associated with non-dipper status, and the combined use of EASIX and PWV improved predictive performance. EASIX may serve as a simple marker for early risk stratification; however, larger prospective studies are needed.

## Introduction

Hypertension represents one of the most common chronic conditions worldwide and continues to be a major contributor to cardiovascular morbidity and mortality [1]. Although diagnostic techniques and therapeutic options have improved considerably over recent decades, effective blood pressure (BP) control remains insufficient in a large proportion of patients [1]. In daily clinical practice, treatment decisions are still largely guided by office-based or average BP measurements [1]. However, increasing evidence suggests that BP is not a static parameter and follows a circadian pattern, with important prognostic implications related to its day–night variability [1, 2]. In this context, under normal physiological conditions, BP decreases during sleep as a result of reduced sympathetic activity and lower vascular tone. A nocturnal decline of more than 10% compared with daytime values is generally defined as a “dipping” pattern, whereas a smaller reduction or the absence of such decline characterizes a “non-dipper” profile. This altered circadian rhythm is now regarded as a clinically relevant phenotype rather than a simple measurement artifact. Numerous studies have demonstrated that non-dipper status is associated with unfavorable cardiovascular and renal outcomes, independent of average BP levels.

Patients with non-dipper hypertension have been shown to exhibit a higher burden of endothelial dysfunction, target organ damage, including left ventricular hypertrophy, impaired renal function, and structural vascular changes [2–6]. Furthermore, this abnormal BP pattern has been linked to an increased risk of stroke, myocardial infarction, and overall mortality [2, 4–6]. These observations emphasize the importance of nocturnal BP regulation and have stimulated growing interest in clarifying the underlying mechanisms responsible for disturbed circadian BP profiles [7]. To this end, the development of non-dipper hypertension is considered to be multifactorial, involving alterations in autonomic regulation, endothelial dysfunction, chronic inflammation, and oxidative stress [8, 9]. Accordingly, the identification of novel laboratory-derived biomarkers has become a key focus. In line with these mechanisms, recent studies have explored indices such as the uric acid-to-albumin ratio to identify non-dipper patterns [10]. Beyond these metabolic markers, structural and functional vascular changes also play a critical role; among these, arterial stiffness plays a central role [11]. Increased rigidity of the arterial wall may compromise baroreflex function and reduce vascular compliance, thereby limiting the normal nocturnal BP decline [12]. For this reason, the assessment of arterial stiffness has gained importance in studies investigating abnormal circadian BP patterns [12].

Pulse wave velocity (PWV) is widely recognized as the reference noninvasive method for evaluating arterial stiffness and has been shown to predict cardiovascular events and mortality independently [13, 14]. Previous investigations have reported higher PWV values in non-dipper hypertensive patients compared with dipper individuals [15]. Nevertheless, the relationship between arterial stiffness and laboratory markers reflecting endothelial injury and systemic inflammation remains incompletely understood, particularly in newly diagnosed and untreated patients, in whom the influence of antihypertensive therapy is absent.

The Endothelial Activation and Stress Index (EASIX) is a composite score derived from lactate dehydrogenase (LDH), serum creatinine, and platelet count [16]. Originally developed as a prognostic indicator in hematological and transplantation-related conditions, the EASIX has more recently gained attention as a potential marker of endothelial injury, microvascular impairment, and cellular stress in cardiovascular disorders [17–19]. Lactate dehydrogenase reflects ongoing tissue damage and increased cellular turnover [20], while serum creatinine provides information on renal function and microvascular integrity [21]. In addition, platelet count is closely associated with endothelial activation and thrombo-inflammatory activity [22]. When considered together, these routinely measured parameters offer a comprehensive assessment of vascular and endothelial stress. Emerging data suggest that EASIX may be associated with clinical outcomes in several cardiovascular and systemic conditions [17–19]. However, its relationship with circadian BP patterns has not been adequately explored. Moreover, limited information is available regarding the combined assessment of EASIX and arterial stiffness in treatment-naïve hypertensive populations. This lack of comprehensive evidence represents an important gap in the current literature.

Therefore, the present study was designed to examine the association between nocturnal dipping status, EASIX score, and arterial stiffness assessed by PWV in newly diagnosed hypertensive patients who had not received antihypertensive treatment. By focusing on untreated individuals, we aimed to reduce potential confounding effects related to medication use. We hypothesized that patients with a non-dipper pattern would exhibit higher EASIX values and greater arterial stiffness than dipper patients, reflecting increased endothelial stress and vascular dysfunction.

## Methods

### Study design and study population

This retrospective observational study was conducted at Selçuk University Faculty of Medicine and included consecutive patients who underwent 24-h ambulatory blood pressure monitoring (ABPM) for the initial assessment of newly diagnosed hypertension between January 2020 and November 2025. The study protocol complied with the principles outlined in the Declaration of Helsinki and was approved by the Ethics Committee of Selçuk University Faculty of Medicine (Approval Number: 2025/612). Given the retrospective design, the requirement for written informed consent was waived. To reduce the risk of selection bias, all eligible patients who met the predefined criteria during the study period were consecutively screened and enrolled, without the use of random sampling procedures.

Patients were included if they had newly diagnosed essential hypertension in accordance with current European Society of Cardiology (ESC) guidelines and had not received any antihypertensive treatment at the time of ABPM [1]. The diagnosis of hypertension was established based on ABPM criteria [1].

Exclusion criteria included secondary hypertension; known coronary artery disease; recent acute coronary syndrome, percutaneous coronary intervention, or coronary artery bypass graft surgery within the preceding three months; heart failure; diabetes mellitus; chronic kidney disease or moderate renal dysfunction (estimated glomerular filtration rate <60 mL/min/1.73 m²); familial hyperlipidemia or ongoing lipid-lowering therapy; active or chronic inflammatory or infectious diseases; known malignancy; significant valvular heart disease; left ventricular ejection fraction below 40%; anemia (hemoglobin <13 g/dL in men and <12 g/dL in women); hematologic disorders; history of blood transfusion within the previous three months; and age above 75 years. Patients with incomplete ambulatory blood pressure monitoring recordings or missing laboratory data required for calculation of the EASIX were also excluded. These criteria were applied to minimize the potential confounding effects of comorbid conditions and recent cardiovascular events on inflammatory markers, endothelial function, lipid metabolism, arterial stiffness, and laboratory parameters.

### Ambulatory blood pressure monitoring and dipping classification

Twenty-four-hour ABPM was performed using a validated oscillometric device (Mobil-O-Graph, I.E.M. GmbH, Stolberg, Germany). Measurements were obtained from the non-dominant arm with an appropriately sized cuff. Blood pressure recordings were scheduled at 15-min intervals during daytime hours (07:00–22:00) and at 30-min intervals during nighttime hours (22:00–07:00).

Participants were advised to maintain their usual daily activities and to keep their arm relaxed and immobile during measurements. In accordance with current recommendations, only recordings with at least 70% valid measurements, including a minimum of 20 daytime and 7 nighttime readings, were considered suitable for analysis. Recordings not meeting these quality standards were excluded.

Hypertension was defined as a 24-h mean systolic blood pressure of at least 130 mmHg and/or a diastolic blood pressure of at least 80 mmHg, in line with ESC guidelines [1]. Mean daytime and nighttime systolic blood pressure values were calculated from the recorded measurements.

The percentage of nocturnal systolic blood pressure decline was calculated using the following formula [1]:

Nocturnal SBP decline (%) = [(Daytime SBP − Nighttime SBP) / Daytime SBP] × 100

A nocturnal systolic reduction greater than 10% was classified as a dipper pattern, whereas a reduction of 10% or less was defined as a non-dipper pattern. Dipping status was primarily determined on the basis of systolic blood pressure, with diastolic values considered supportive.

### Assessment of arterial stiffness

Arterial stiffness was evaluated by measuring PWV using the Mobil-O-Graph system. PWV values were automatically derived through pulse wave analysis based on proprietary algorithms integrated into the device software.

The Mobil-O-Graph has previously been validated against invasive and tonometric reference techniques and has demonstrated acceptable accuracy in both clinical and epidemiological settings [13, 14]. All PWV measurements obtained during ABPM were recorded and included in the subsequent analyses.

### Laboratory measurements and EASIX calculation

Venous blood samples were collected after an overnight fasting period of at least eight hours at the time of ABPM referral. Complete blood count parameters, including white blood cell count, neutrophil count, lymphocyte count, monocyte count, hemoglobin concentration, and platelet count, were determined using automated hematology analyzers.

Serum biochemical analyses included fasting plasma glucose, creatinine, total cholesterol, triglycerides, low-density and high-density lipoprotein cholesterol, electrolytes, uric acid, thyroid-stimulating hormone, serum albumin, and lactate dehydrogenase. All measurements were performed using standardized enzymatic methods in the central laboratory.

The EASIX was calculated for each participant according to the following formula [18]:

EASIX = [LDH (U/L) × creatinine (mg/dL)] / platelet count (10⁹/L)

### Statistical analysis

Statistical analyses were performed using SPSS software (version 22.0; IBM Corp., Armonk, NY, USA). The normality of continuous variables was assessed using the Kolmogorov–Smirnov test. Continuous variables are presented as mean ± standard deviation or median with interquartile range, while categorical variables are expressed as frequencies and percentages.

The primary aim of the statistical analysis was to evaluate the association between EASIX as the main exposure variable and nocturnal dipping status as the clinical outcome.

Baseline demographic, clinical, and laboratory characteristics were compared between dipper and non-dipper groups using independent-samples t-tests or Mann–Whitney U tests for continuous variables and chi-square or Fisher’s exact tests for categorical variables, as appropriate.

Binary logistic regression analyses were performed to identify factors associated with non-dipper status. Variables with a p value < 0.20 in univariate analyses were entered into the multivariate model. EASIX was included as the primary exposure variable and was standardized as a Z-score to allow interpretation per one standard deviation increase. To avoid multicollinearity, individual components of the EASIX score were not included separately in the regression models.

PWV was analyzed as a continuous marker of arterial stiffness and compared between dipper and non-dipper groups. The relationship between EASIX and PWV was additionally evaluated using Spearman correlation analysis.

A combined predictive model for non-dipper status was constructed using binary logistic regression including both EASIX and PWV as independent variables. The discriminative performance of this model, as well as of EASIX and PWV individually, was assessed using receiver operating characteristic (ROC) curve analysis. The optimal cutoff value for EASIX was determined using the Youden index.

All statistical tests were two-sided, and a p value < 0.05 was considered statistically significant.

## Results

### Baseline demographic and clinical characteristics

A total of 163 newly diagnosed hypertensive patients were included in the final analysis, of whom 72 were classified as non-dippers and 91 as dippers. Baseline demographic and clinical characteristics according to dipping status are summarized in Table 1. No significant differences were observed between the two groups with respect to age, sex distribution, body mass index, or smoking status (all p > 0.05). The mean age was 50.53 ± 16.99 years in the non-dipper group and 50.34 ± 15.43 years in the dipper group (p = 0.941). Likewise, the proportions of male participants and current smokers were comparable between groups.

**Table 1:** Demographic and Clinical Characteristics According to Dipping Status.

Variable	Non-dipper (n = 72)	Dipper (n = 91)	P Value
Age, years	50.53 ± 16.99	50.34 ± 15.43	0.941
Gender, male, n (%)	32 (44.4)	42 (46.2)	0.875
BMI, kg/m^2^	27.72 (25.05-31.21)	27.47 (25.61-31.35)	0.556
Smoking, n (%)	10 (13.9)	20 (22)	0.224

*BMI* body mass index.

### Laboratory findings and EASIX scores

Laboratory findings and EASIX scores stratified by dipping status are presented in Table 2. Most hematological and biochemical parameters, including white blood cell count, hemoglobin, platelet count, fasting plasma glucose, lipid profile, creatinine, blood urea nitrogen, sodium, calcium, phosphorus, uric acid, and thyroid-stimulating hormone, did not differ significantly between non-dipper and dipper patients. In contrast, serum potassium levels were significantly lower in the non-dipper group (4.27 ± 0.32 vs. 4.40 ± 0.31 mmol/L, p = 0.007), whereas lactate dehydrogenase levels were significantly higher (197 [171–223] vs. 192 [163–217] U/L, p = 0.004). In addition, median EASIX values were markedly elevated among non-dipper patients compared with dipper individuals (0.73 [0.50–0.99] vs. 0.52 [0.39–0.70], p < 0.001).

**Table 2:** Laboratory Parameters and EASIX Score According to Dipping Status.

Variable	Non-dipper (n = 72)	Dipper (n = 91)	P Value
WBC Count, x10⁹/L	6.85 (5.88-8.25)	6.90 (5.76-8.22)	0.363
Hemoglobin, g/dL	14.00 (13.00-15.05)	13.90 (13.12-15.25)	0.231
Platelet count, x10⁹/L	254 (207-329)	269 (231-313)	0.273
FBG, mg/dL	88.50 (82-96)	94 (87-105)	0.826
Total cholesterol, mg/dL	189 (169-234)	191 (160-222)	0.709
LDL cholesterol, mg/dL	117 (98-151)	112 (93-142)	0.093
HDL cholesterol, mg/dL	45 (37-54)	47 (41-56)	0.107
Triglycerides, mg/dL	134 (88-206)	127 (95-181)	0.561
Creatinine, mg/dL	0.80 (0.69-0.95)	0.81 (0.70-0.89)	0.515
BUN, mg/dL	27 (24-35)	28 (23-36)	0.799
Sodium, mEq/L	140 (138-141)	139 (138-141)	0.892
Potassium, mmol/L	4.27 ± 0.32	4.40 ± 0.31	0.007
Calcium, mg/dL	9.40 (9.10-9.72)	9.40 (9.10-9.67)	0.191
Phosphorus, mg/dL	3.49 (3.25-3.90)	3.50 (3.20-3.80)	0.382
Uric acid, mg/dL	5.10 (4.17-6.50)	4.80 (4.00-6.57)	0.957
TSH, µIU/ml	1.90 (1.53-2.80)	2.03 (1.40-2.71)	0.299
Lactate dehydrogenase, U/L	197 (171-223)	192 (163-217)	0.004
EASIX score	0.73 (0.50-0.99)	0.52 (0.39-0.70)	<0.001

*BUN* blood urea nitrogen, *EASIX* endothelial activation and stress index, *FBG* fasting blood glucose, *HDL* high-density lipoprotein, *LDL* low-density lipoprotein, *TSH* thyroid stimulating hormone, *WBC* white blood cells.

### Ambulatory blood pressure parameters and arterial stiffness

Ambulatory blood pressure and arterial stiffness parameters are shown in Table 3. Twenty-four-hour systolic blood pressure values were similar between the two groups (p = 0.933). However, 24-h diastolic blood pressure was significantly lower in non-dipper patients (91 [84–102] vs. 96 [88–102] mmHg, p = 0.019). Daytime systolic and diastolic blood pressure values were also significantly lower in the non-dipper group (p < 0.001 and p = 0.003, respectively). As expected, nighttime systolic blood pressure was significantly higher in non-dipper patients than in dipper patients (143 [137–147] vs. 130 [125–135] mmHg, p < 0.001), whereas nighttime diastolic blood pressure did not differ significantly between groups. Pulse wave velocity was significantly greater in the non-dipper group (7.92 ± 1.97 vs. 7.05 ± 1.69 m/s, p = 0.003), indicating increased arterial stiffness.

**Table 3:** Ambulatory Blood Pressure Monitoring and Arterial Stiffness Parameters.

Variable	Non-dipper (n = 72)	Dipper (n = 91)	P Value
24 h SBP, mmHg	144 (139-152)	147 (137-153)	0.933
24 h DBP, mmHg	91 (84-102)	96 (88-102)	0.019
Daytime SBP, mmHg	142 (139-150)	148 (142-154)	<0.001
Daytime DBP, mmHg	92 (85-102)	97 (90-104)	0.003
Nighttime SBP, mmHg	143 (137-147)	130 (125-135)	<0.001
Nighttime DBP, mmHg	89 (81-100)	86 (81-98)	0.981
PWV, m/s	7.92 ± 1.97	7.05 ± 1.69	0.003

DBP, diastolic blood pressure; PWV, pulse wave velocity; SBP, systolic blood pressure.

### Factors independently associated with non-dipper hypertension

The results of univariate and multivariate logistic regression analyses are summarized in Table 4. In univariate analysis, serum potassium, lactate dehydrogenase, standardized EASIX score, and PWV were significantly associated with non-dipper status. Although low-density lipoprotein cholesterol and calcium levels did not reach statistical significance, both variables demonstrated p values below 0.20 and were therefore included in the multivariate model.

**Table 4:** Binary Logistic Regression Analysis of Factors Associated with Non-Dipper Hypertension.

	Univariate Analysis		Multivariate Analysis	
Variable	OR (95% CI)	P Value	OR (95% CI)	P Value
Age	1.001 (0.982-1.020)	0.941	-	-
BMI, kg/m^2^	0.965 (0.906-1.028)	0.265	-	-
Platelet count, 10⁹/L	0.998 (0.994-1.002)	0.331	-	-
Total cholesterol, mg/dL	1.003 (0.995-1.010)	0.468	-	-
LDL cholesterol, mg/dL	1.008 (1.000-1.017)	0.056*	1.006 (0.995-1.017)	0.286
Creatinine, mg/dL	0.548 (0.099-3.023)	0.490	-	-
Potassium, mmol/L	0.251 (0.089-0.703)	0.009*	0.331 (0.093-1.183)	0.089
Calcium, mg/dL	0.559 (0.283-1.105)	0.094*	0.964 (0.427-2.175)	0.929
Lactate dehydrogenase, U/L	1.013 (1.004-1.023)	0.003	-	-
EASIX (per 1-SD increase)	2.820 (1.801-4.415)	<0.001*	2.553 (1.568-4.156)	<0.001*
PWV, m/s	1.294 (1.086-1.541)	0.004*	1.358 (1.090-1.691)	0.006*

*BMI* body mass index, *EASIX* endothelial activation and stress index, *LDL* low density lipoprotein, *PWV* pulse wave velocity.

*Variables with p < 0.20 in univariate analysis were included in the multivariate model.

Serum potassium showed a significant inverse association with non-dipper status in univariate analysis (OR: 0.251, 95% CI: 0.089–0.703, p = 0.009). After adjustment for potential confounders, this association was attenuated and no longer statistically significant, although a borderline trend persisted (OR: 0.331, 95% CI: 0.093–1.183, p = 0.089).

In the multivariate model, standardized EASIX score and PWV remained independently associated with non-dipper status. Each one standard deviation increase in EASIX was associated with a 2.55-fold higher likelihood of non-dipper hypertension (OR: 2.553, 95% CI: 1.568–4.156, p < 0.001). Similarly, higher PWV values were independently related to a non-dipper pattern (OR: 1.358, 95% CI: 1.090–1.691, p = 0.006). LDL cholesterol and calcium did not retain statistical significance in the adjusted model.

### ROC Curve analysis of EASIX and PWV

A Spearman correlation analysis revealed a statistically significant positive correlation between EASIX and PWV (r = 0.165, p = 0.036).

**Fig. 1:**
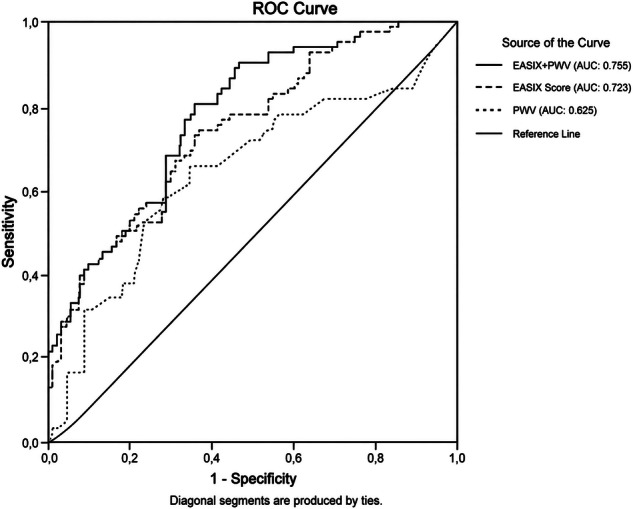
Comparison of Receiver Operating Characteristic (ROC) curves for the prediction of non-dipper status. The combined model (EASIX + PWV), represented by the blue line, demonstrated superior diagnostic performance with an area under the curve (AUC) of 0.755 (95% CI: 0.682–0.828, p < 0.001). The individual AUC values for EASIX and PWV were 0.723 (95% CI: 0.646–0.800, p < 0.001) and 0.625 (95% CI: 0.536–0.715, p = 0.006), respectively. An EASIX cutoff value of >0.597 yielded a sensitivity of 68.1% and a specificity of 64.8% (Youden index).

Receiver operating characteristic (ROC) curve analysis was performed to evaluate the performance of EASIX, PWV, and their combination in discriminating nocturnal non-dipping status (Fig. 1). The area under the curve (AUC) was 0.723 (95% CI: 0.646–0.800, p < 0.001) for EASIX and 0.625 (95% CI: 0.536–0.715, p = 0.006) for PWV. The combined model (EASIX + PWV) demonstrated higher discriminative ability, with an AUC of 0.755 (95% CI: 0.682–0.828, p < 0.001). An optimal EASIX cutoff value of >0.597 yielded a sensitivity of 68.1% and a specificity of 64.8%, according to the Youden index. Consistent with these findings, a combined logistic regression model including both EASIX and PWV demonstrated that these variables provide complementary contributions to the prediction of non-dipper status.

## Discussion

To our knowledge, limited data are available regarding the relationship between EASIX, arterial stiffness, and nocturnal blood pressure patterns in newly diagnosed and untreated hypertensive patients. In this context, we examined the relationships between circadian blood pressure patterns, endothelial stress, and arterial stiffness in newly diagnosed and untreated individuals. Our main finding was that both EASIX score and pulse wave velocity were significantly higher in non-dipper patients and remained independently associated with non-dipper status after multivariate adjustment. These findings suggest that increased endothelial stress and vascular stiffness are closely related to impaired nocturnal blood pressure decline in the early stages of hypertension, further supporting the concept that the non-dipper profile represents a high-risk phenotype even before the initiation of antihypertensive therapy.

Arterial stiffness is considered an important factor in abnormal circadian blood pressure regulation [11]. In our cohort, PWV was significantly higher in non-dipper patients, an association that was also supported by ROC analysis demonstrating significant discriminatory power. Notably, the combined model of EASIX and PWV yielded a superior area under the curve (AUC: 0.755), suggesting a complementary and additive contribution of endothelial stress and vascular stiffness in identifying non-dipper status. Increased arterial rigidity may be associated with impaired baroreceptor sensitivity and reduced vascular compliance, which could contribute to sustained nocturnal blood pressure levels [23].

Recent evidence suggests that the EASIX may have clinical relevance in different cardiovascular settings, including coronary artery disease and heart failure [18, 24–26]. Our findings are consistent with recent data demonstrating that EASIX shows good performance in identifying inadequate blood pressure control [27]. EASIX, as an established marker of endothelial stress, may indicate multiple aspects of cellular injury, renal function, and thrombo-inflammation. In the present study, higher EASIX values in non-dipper patients may therefore indicate increased endothelial stress and microvascular dysfunction. While PWV reflects structural arterial remodeling, EASIX may indicate ongoing inflammatory and cellular stress, suggesting that these two markers represent complementary components of vascular pathology [18, 26, 28].

A notable finding in our univariate analysis was the significantly lower serum potassium levels in non-dipper patients compared to dippers. Potassium plays a pivotal role in vascular homeostasis by promoting endothelium-dependent vasodilation through the hyperpolarization of vascular smooth muscle cells and the activation of Na⁺/K⁺-ATPase pumps [29, 30]. Beyond its direct vasodilatory effects, potassium is known to reduce oxidative stress and inhibit platelet aggregation, thereby preserving endothelial integrity [29]. From a pathophysiological perspective, lower potassium levels may reflect increased activity of the renin–angiotensin–aldosterone system, particularly elevated aldosterone levels, which have been associated with impaired nocturnal blood pressure decline and the non-dipper pattern [31]. Lower potassium levels have also been associated with disturbed circadian blood pressure rhythms, particularly in overweight or obese individuals [32]. In our study, although potassium was significantly associated with the non-dipper pattern in univariate analysis, this relationship did not persist after multivariate adjustment. This suggests that although potassium levels are associated with circadian BP regulation, their effect may be mediated by or overlap with broader markers of vascular stress such as PWV and the EASIX score. Similarly, lactate dehydrogenase was associated with non-dipper status in univariate analysis but was excluded from multivariate analyses due to its inclusion in the EASIX calculation.

An important strength of this study is the inclusion of treatment-naïve patients, which minimizes the potential influence of antihypertensive medications on blood pressure patterns and laboratory parameters. However, several limitations should be acknowledged. First, the retrospective, single-center design and moderate sample size may limit causal inference and generalizability. Second, dipping status was determined based on a single 24-h ABPM recording, which may limit reproducibility and lead to potential misclassification due to day-to-day variability. Furthermore, we primarily categorized patients as dippers or non-dippers without sub-classifying reverse or extreme dipping patterns. Regarding arterial stiffness, although the Mobil-O-Graph is a validated device, it provides an estimated PWV based on oscillometric algorithms and does not directly measure carotid-femoral PWV, which is considered the gold standard. Additionally, information on sleep quality and obstructive sleep apnea was not available, and the absence of longitudinal follow-up prevents the assessment of long-term prognostic implications. Residual confounding cannot be excluded. While the combined model showed promising performance, its clinical utility requires validation in larger prospective and interventional studies.

## Conclusion

In conclusion, this study demonstrates that non-dipper hypertension in newly diagnosed and untreated patients is associated with higher EASIX scores and increased arterial stiffness, even in the early stages of hypertension. Both parameters were independently related to non-dipper status, suggesting that endothelial activation and vascular dysfunction are closely linked to abnormal nocturnal blood pressure regulation. These findings highlight the potential of EASIX as a simple and accessible tool for early risk stratification. Further longitudinal and interventional studies are warranted to clarify the underlying mechanisms and the long-term clinical significance of these associations.

## Summary

### What is known about the topic

- Non-dipper hypertension is associated with increased cardiovascular risk, endothelial dysfunction, and target organ damage.
- Increased arterial stiffness, measured by pulse wave velocity, is related to impaired nocturnal blood pressure decline.
- Endothelial dysfunction and inflammation play an important role in abnormal circadian blood pressure patterns.

### What this study adds

- This study shows that EASIX, calculated from lactate dehydrogenase, creatinine, and platelet count, is independently associated with non-dipper status in newly diagnosed and untreated hypertensive patients.
- Higher EASIX values and increased pulse wave velocity reflect increased endothelial stress and vascular dysfunction in non-dipper patients.
- Since EASIX is based on routinely available laboratory parameters, it may be a simple and low-cost marker for early risk stratification.

## Data Availability

The data presented in this study are available on reasonable request from the corresponding author. The data are not publicly available due to institutional and ethical restrictions.
