# Correction: EASIX and Arterial stiffness in relation to nocturnal blood pressure patterns in newly diagnosed hypertension

**DOI:** 10.1038/s41371-026-01167-0

**Published:** 2026-06-01

**Authors:** Muhammed Ulvi Yalcin, Kadri Murat Gurses, Halil Ozalp, Muhammet Salih Ates, Abdullah Tuncez, Huseyin Tezcan, Yasin Ozen, Tugay Dedebali, Mustafa Abdullah Kebude, Bulent Behlul Altunkeser

**Affiliations:** 1 Department of Cardiology, Selcuk University Faculty of Medicine, Konya, Turkey; 2 Department of Cardiology, Ahi Evran University Faculty of Medicine, Kırsehir, Turkey

**Keywords:** Prognosis, Risk factors

Correction to: *Journal of Human Hypertension* 10.1038/s41371-026-01160-7, published online 19 May 2026

In this article the author’s name Yasin Ozen was incorrectly written as Yasin Ozer.

In this article the affiliation details for Muhammet Salih Ates were incorrectly given as ‘Department of Cardiology, Ahi Evren University Faculty of Medicine, Ankara, Turkey’ but should have been ‘Department of Cardiology, Ahi Evran University Faculty of Medicine, Kırsehir, Turkey’.

The original article has been corrected.

